# Body mass index in early and middle-late adulthood and risk of localised, advanced and fatal prostate cancer: a population-based prospective study

**DOI:** 10.1038/bjc.2011.319

**Published:** 2011-08-16

**Authors:** A Discacciati, N Orsini, S-O Andersson, O Andrén, J-E Johansson, A Wolk

**Affiliations:** 1 Division of Nutritional Epidemiology, Institute of Environmental Medicine, Karolinska Institutet, Box 210, 171 77, Stockholm, Sweden; 2 Department of Urology, Örebro University Hospital, Örebro, 701 85, Sweden

**Keywords:** prostate cancer, body mass index, obesity, body size, prospective cohort study

## Abstract

**Background:**

The relationships between body mass index (BMI) during early and middle-late adulthood and incidence of prostate cancer (PCa) by subtype of the disease (localised, advanced) and fatal PCa is unclear.

**Methods:**

A population-based cohort of 36 959 Swedish men aged 45–79 years was followed up from January 1998 through December 2008 for incidence of PCa (1530 localised and 554 advanced cases were diagnosed) and through December 2007 for PCa mortality (225 fatal cases).

**Results:**

From a competing-risks analysis, incidence of localised PCa was observed to be inversely associated with BMI at baseline (middle-late adulthood; rate ratio (RR) for 35 kg m^–2^ when compared with 22 kg m^–2^ was 0.69 (95% CI 0.52–0.92)), but not at age 30. For fatal PCa, BMI at baseline was associated with a nonstatistically significant increased risk (RR for every five-unit increase: 1.12 (0.88–1.43)) and BMI at age 30 with a decreased risk (RR for every five-unit increase: 0.72 (0.51–1.01)).

**Conclusion:**

Our results indicate an inverse association between obesity during middle-late, but not early adulthood, and localised PCa. They also suggest a dual association between BMI and fatal PCa – a decreased risk among men who were obese during early adulthood and an increased risk among those who were obese during middle-late adulthood.

The aetiology of prostate cancer (PCa), the most common cancer malignancy in men in the developed countries and the second most common one worldwide (Jemal *et al*, 2011), is still largely unknown (Gronberg, 2003). It seems nonetheless to differ depending on the subtype of the disease at the time of diagnosis, namely localised and advanced PCa cases (Hsing *et al*, 2007).

Body adiposity is related to both sex hormones and insulin-like growth factor I (IGF-I) (Zumoff, 1988; Pasquali *et al*, 1991; Yamamoto and Kato, 1993; Kaaks *et al*, 2000; Lima *et al*, 2000; Lukanova *et al*, 2002). Owing to possible relationships between sex hormones and PCa (Platz *et al*, 2005; Severi *et al*, 2006) and between IGF-I and PCa (Roddam *et al*, 2008), body mass index (BMI) has itself been studied as a potential risk factor (Freedland and Platz, 2007). The results, however, are inconsistent.

For incidence of localised PCa, four studies observed a statistically significant inverse relationship with BMI (Gong *et al*, 2006; Littman *et al*, 2007; Rodriguez *et al*, 2007; Wright *et al*, 2007), seven studies observed nonstatistically significant association (Schuurman *et al*, 2000; MacInnis *et al*, 2003; Kurahashi *et al*, 2006; Giovannucci *et al*, 2007; Pischon *et al*, 2008; Wallstrom *et al*, 2009; Stocks *et al*, 2010) and two studies with a limited number of cases observed a suggestion of direct association, although nonstatistically significant (Cerhan *et al*, 1997; Putnam *et al*, 2000).

For incidence of advanced PCa, three studies observed a statistically significant direct association with BMI (Gong *et al*, 2006; Giovannucci *et al*, 2007; Rodriguez *et al*, 2007), six observed no association (Schuurman *et al*, 2000; Littman *et al*, 2007; Wright *et al*, 2007; Pischon *et al*, 2008; Wallstrom *et al*, 2009; Stocks *et al*, 2010), and among four other studies with a small number of cases, two observed a statistically significant direct association (Putnam *et al*, 2000; MacInnis *et al*, 2003), but not others (Cerhan *et al*, 1997; Kurahashi *et al*, 2006).

To the best of our knowledge, only one study adjusted the multivariable models for BMI during early adulthood when examining the possible relationships between BMI during middle-late adulthood and risk of localised and advanced PCa (Giovannucci *et al*, 2007).

BMI during middle-late adulthood as well as in earlier stages of life could be critical for the development of PCa (Hsing, 1996; Giovannucci *et al*, 1997; Gapstur *et al*, 2002). However, only a limited number of prospective studies examined the relationship between BMI during early adulthood and incidence of PCa by subtype of the disease. Among the studies of localised PCa, inconsistent results were observed with statistically significant inverse (Wright *et al*, 2007), direct (Schuurman *et al*, 2000) and null associations (Littman *et al*, 2007). Four prospective studies examined the association between BMI in early adulthood and incidence of advanced PCa, observing null (Schuurman *et al*, 2000; Littman *et al*, 2007; Wright *et al*, 2007) and inverse associations (Giovannucci *et al*, 1997).

As the available evidence is limited and the results are inconsistent, the aim of our population-based cohort study was to examine the relationships between BMI during early adulthood (age 30 years) and during middle-late adulthood (age 45–79 years) regarding incidence of localised, advanced and fatal PCa.

## Materials and methods

The population-based cohort of Swedish men was established during 1997 to 1998, when all eligible men (*n*=100 303) aged 45–79 years residing in Västmanland and Örebro counties in central Sweden received an invitation to participate in the study along with a self-administered questionnaire. The questionnaire included questions about current weight, weight at age 30 years, height, educational level, smoking habit, family history of PCa, physical activity and diet. A total of 48 645 men returned the questionnaire.

We excluded participants who returned an incomplete questionnaire (*n*=92), died before 1 January 1998 (*n*=55), had a previous cancer diagnosis (*n*=2592) or had BMI at baseline age or at age 30 <15, >40 kg m^–2^ (*n*=196) or missing (*n*=8751), thus leaving 36 959 subjects available for the analyses. This population-based cohort is representative of Swedish males aged 45–79 years in terms of age distribution, educational level and prevalence of overweight (Norman *et al*, 2002). Incidence rates in 1998 per 100 000 men are also comparable: for example, the incidence rate among men aged 65–69 years is 603 in our cohort and 595 in the entire Sweden (NBHW, 2000; Orsini *et al*, 2009).

The BMI is a surrogate measurement for body adiposity as it is highly correlated with the percentage of body fat calculated hydrostatically by body submersion (Revicki and Israel, 1986) and is calculated as weight in kilograms divided by height in metres squared (kg m^–2^). Validity of BMI estimates based on self-reported weight and height as measured by a slope of linear regression was 0.89 in the Swedish adult population (Kuskowska-Wolk *et al*, 1989).

Information about usual physical activity levels during the previous year was collected using five questions relating to occupation, housework, walking or cycling, leisure-time exercise and inactive leisure time. To calculate the activity score of the specific activity type, the intensity of these activities (expressed as MET-h) was multiplied by the reported time in hours. Based on questionnaire data we estimated a total activity score (expressed as MET-h per day) by adding the single activity scores (Norman *et al*, 2001).

Incident cases of PCa were ascertained by computerised record linkage with the Swedish National Cancer Register and the Regional Cancer Register covering the study area, both of which are estimated to be almost 100% complete (Mattsson and Wallgren, 1984). Information on tumour–node–metastasis (TNM) stage, Gleason grade and value of prostate-specific antigen (PSA) at PCa diagnosis were available from the Swedish Prostate Cancer Quality Registry. Incident cases were classified by subtype as localised (T1–2 and NX-0 and (MX-0 or PSA <20 ng ml^–1^ or Gleason grade ⩽7)) or advanced (T3–4 and NX-1 and (MX-1 or PSA >100 ng ml^–1^ or Gleason grade >7)).

Information on fatal PCa cases was ascertained through linkage to the Swedish Register of Death Causes at the National Board of Health and Welfare. Classification of deaths was based on International Classification of Diseases (ICD-10, code 61 for PCa).

From 1 January 1998 to 31 December 2008, during 371 792 person-years, we documented 2336 incident cases of PCa. Of these, 1530 were classified as localised and 554 as advanced cases. From 1 January 1998 to 31 of December 2007, during 333 702 person-years, we documented 225 cases of fatal PCa.

### Statistical analysis

The Cox proportional hazards model was used to estimate PCa incidence rate ratios (RRs) and 95% Wald confidence intervals associated with BMI at baseline age and at age 30 years. Each subject accrued follow-up time from 1 January 1998 until the date of PCa diagnosis, death from any cause or study end (31 December 2008), whichever came first. For fatal PCa analysis, each participant accrued follow-up time from 1 January 1998 until the date of PCa death, death from any cause or study end (31 December 2007), whichever came first.

We categorised BMI at baseline age in six predefined groups (<21, 21–22.9, 23–24.9, 25–27.4, 27.5–29.9 or ⩾30 kg m^–2^) in order to investigate the study participants’ characteristics at baseline.

We calculated the RRs from the Cox proportional hazards models for the BMI at baseline age and BMI at age 30 years in correspondence with the midpoints of the aforementioned categories: 18, 22 (reference), 24, 26.25, 28.75 and 35 kg m^–2^ to present results in a tabular form. This reference was chosen because in a pooled analysis of 1.46 million white adults, the lowest risk of cancer mortality was observed in the BMI category between 20.0 and 22.5 kg m^–2^ (Berrington de Gonzalez *et al*, 2010).

BMI values at baseline age and at age 30 years were mutually adjusted in both the age-adjusted and multivariable models and were modelled as continuous variables using fractional polynomials (Royston *et al*, 1999) whenever this provided a better overall fit of the model calculated using the Akaike information criterion (AIC) (Akaike, 1974). All the multivariable analyses were adjusted for baseline age (years), total energy intake (kcal), total physical activity (<37.9, 38–40.9, 41–44.9, ⩾45 MET-h per day or missing), years of education (1–9, 9–12 or >12 years), smoking status (current, former or never smoker), family history of PCa (yes, no or don’t know) and personal history of diabetes (yes or no).

We checked whether the proportional hazard assumption was reasonable by means of scaled Schoenfeld's residuals, which were regressed against the natural logarithm of the survival time. There was no evidence of departure from this assumption.

It is well known that BMI is associated with overall mortality (Berrington de Gonzalez *et al*, 2010). Therefore, we performed a sensitivity analysis using the competing-risks regression, where all the deaths from other causes than PCa were considered as competing events. This analysis allowed us to evaluate a potential effect of competing risks on the observed results (Fine and Gray, 1999).

All reported *P*-values are two sided. All statistical analyses were performed with Stata release 11 (StataCorp, College Station, TX, USA).

## Results

Age-standardised baseline characteristics by category of BMI at baseline age of the 36 959 study participants are shown in Table 1. Mean age and mean total energy intake did not change significantly across the six levels of BMI at baseline age, as well as prevalence of subjects with family history of PCa. Higher levels of BMI at baseline age were associated with higher values of BMI at age 30 years (Pearson's correlation coefficient=0.6). Compared with men in the lowest group of BMI at baseline age, those in the higher groups were more likely to have a personal history of diabetes and less likely to be physically active, well-educated or current smokers.

Mean age at PCa diagnosis for localised and advanced cases was 69 and 74 years, respectively. Mean age at death from PCa was 75 years.

Age-adjusted and multivariable RRs for PCa incidence of localised, advanced and fatal PCa in the study population according to BMI levels at baseline age and at age 30 years are presented in Tables 2 and 3, respectively.

For localised PCa we observed in the age-adjusted model a left-skewed ‘inverse U’-shaped relationship with BMI at baseline age. Further adjustment for potential confounders did not substantially change the shape of the relationship. In correspondence with BMI level at baseline age of 35 kg m^–2^, the multivariable model showed a decreased incidence of 29% (6–47%) compared with that at the reference value (22 kg m^–2^). No statistically significant association was observed between BMI at age 30 years and incidence of localised PCa, with a decreased risk of 2% (−13 to 12%) for every five-unit increment. By modelling BMI at baseline age as a fractional polynomial and BMI at age 30 years linearly, the global fit of the model slightly improved with respect to the model, with both variables modelled in a linear fashion (AIC=36 616 *vs* AIC=36 614).

For advanced PCa a direct but statistically nonsignificant association was observed with BMI at baseline age as well as BMI at age 30; in the multivariable model, the RR for BMI at baseline age increased linearly by 4% (−12 to 22%), whereas the RR for BMI at age 30 years decreased linearly by 10% (−27 to 11%), both for every five-unit increment.

For fatal PCa we observed in the multivariable analysis a nonstatistically significant direct association with BMI at baseline age and an inverse linear association with BMI at age 30 years (*P*-value=0.06). For every five-unit increment, the RR for BMI at baseline age increased linearly by 12% (−13 to 43%), whereas the RR for BMI at age 30 years decreased linearly by 27% (−47 to 2%). These results may also suggest a dual effect of BMI on death from PCa, but none of them reached statistical significance. Of the 225 documented cases of fatal PCa, 50 (22%) were classified at diagnosis as localised and 141 (63%) as advanced cases, whereas the remaining 34 cases (15%) were unclassified.

**Figure 1:**
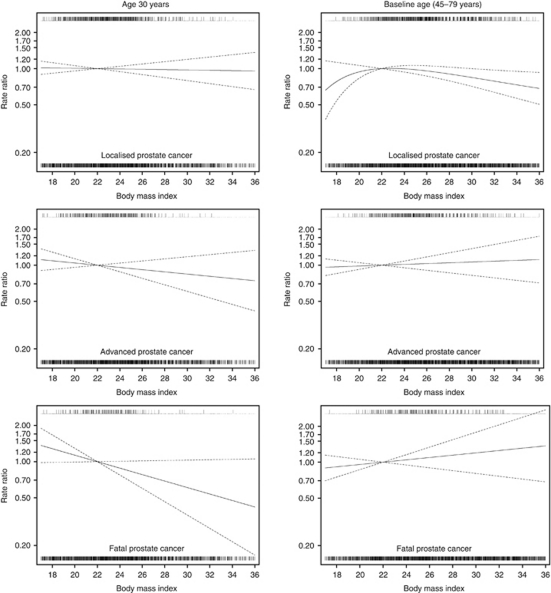
Multivariable rate ratios for BMI at age 30 years and at baseline age as predictors of incidence of localised, advanced and fatal prostate cancer. Data were fitted using a Cox proportional hazards model. BMI values at baseline age (kg m^–2^) and at age 30 years (kg m^–2^) were mutually adjusted and entered as continuous variables into the model; reference value was set at 22 kg m^–2^. Nonlinear relationships were modelled using second-degree fractional polynomials. Data were adjusted for age at baseline (years), total energy intake (kcal), total physical activity (<37.9, 38–40.9, 41–44.9, ⩾45 MET-h per day or missing), years of education (1–9, 9–12 or >12 years), smoking status (current, former or never smoker), family history of prostate cancer (yes, no or don’t know) and personal history of diabetes (yes or no). Dashed lines represent 95% confidence limits. Vertical lines above the curve represent cases of prostate cancer, whereas vertical lines below the curve represent non-cases of prostate cancer.

The relationships between BMI at baseline age and at age 30 years modelled as continuous variables and the incidence of localised, advanced and fatal PCa are displayed graphically in Figure 1.

In order to examine whether preclinical symptoms of cancer might have affected the BMI at baseline age, leading to biased results, we excluded the first 2 years of follow-up from all the analyses. The multivariable results after this exclusion did not appreciably change (data not shown).

Compared with the results obtained using the Cox proportional hazards model, those obtained with the competing-risks regression did not significantly change, but became slightly stronger (Tables 2 and 3).

## Discussion

### BMI during middle-late adulthood and incidence of localised, advanced and fatal PCa

In this population-based prospective cohort study, we observed that high levels of BMI during middle-late adulthood are inversely associated with the incidence of localised PCa. This result is in agreement with some previous prospective studies (Gong *et al*, 2006; Littman *et al*, 2007; Rodriguez *et al*, 2007; Wright *et al*, 2007), but not all (Cerhan *et al*, 1997; Putnam *et al*, 2000; Schuurman *et al*, 2000; MacInnis *et al*, 2003; Kurahashi *et al*, 2006; Giovannucci *et al*, 2007; Pischon *et al*, 2008; Wallstrom *et al*, 2009; Stocks *et al*, 2010). Among those studies where a statistically significant association was not observed, two suggested an inverse association (Schuurman *et al*, 2000; Pischon *et al*, 2008), whereas two studies with a small number of cases suggested a direct association (Cerhan *et al*, 1997; Putnam *et al*, 2000).

In contrast to localised PCa, we observed that high BMI levels during middle-late adulthood were associated with a nonsignificant increased risk of advanced PCa. A statistically significant direct association between BMI during middle-late adulthood and incidence of advanced PCa was observed in some previous prospective studies (Putnam *et al*, 2000; MacInnis *et al*, 2003; Gong *et al*, 2006; Giovannucci *et al*, 2007; Rodriguez *et al*, 2007), but not all (Cerhan *et al*, 1997; Schuurman *et al*, 2000; Kurahashi *et al*, 2006; Littman *et al*, 2007; Wright *et al*, 2007; Pischon *et al*, 2008; Wallstrom *et al*, 2009; Stocks *et al*, 2010).

Our results suggesting an increased risk of fatal PCa are in line with the majority of the previous prospective studies showing a statistically significant positive association between increased BMI during middle-late adulthood and risk of death from PCa (Calle *et al*, 2003; Giovannucci *et al*, 2007; Wright *et al*, 2007; Stocks *et al*, 2010), but not all studies (Rodriguez *et al*, 2001). Similar to the present analysis, in only one previous study the authors adjusted the multivariable analyses also for BMI during early adulthood when examining the relationships between BMI during middle-late adulthood and risk of localised, advanced and fatal PCa (Giovannucci *et al*, 2007).

### BMI during early adulthood and incidence of localised, advanced and fatal PCa

In our study we observed a nonstatistically significant association between BMI during early adulthood (age 30 years) and risk of localised and advanced PCa. Only three prospective studies examined the relationship between BMI during early adulthood and incidence of localised PCa, but they observed inconsistent results: statistically significant direct (Schuurman *et al*, 2000), inverse (Wright *et al*, 2007) and no associations (Littman *et al*, 2007). Four prospective studies examined the association between BMI during early adulthood and incidence of advanced PCa, observing null (Schuurman *et al*, 2000; Littman *et al*, 2007; Wright *et al*, 2007) and inverse associations (Giovannucci *et al*, 1997).

In our study, a weak evidence of an inverse association between BMI at age 30 years and fatal PCa was observed. Our study is the largest one among the previous prospective studies in terms of the number of cases. The existing evidence from prospective studies about a possible association between early-adult BMI and risk of death from PCa is limited, as only three studies with a small number of cases are available. Of these, two studies observed a null relationship with fatal PCa (Wright *et al*, 2007; Burton *et al*, 2010), whereas one observed a direct association (Okasha *et al*, 2002), although nonstatistically significant. A dual effect of obesity is suggested by comparing the observed associations between BMI at age 30 years, BMI at baseline age and incidence of fatal PCa: a decreased risk of fatal PCa among men who were obese during early adulthood and an increased risk among those who were obese during middle-late adulthood.

The inconsistent results in studies regarding BMI during late-adulthood and risk of PCa might be because of complex relationships between obesity and hormones, like testosterone and IGF-I. In particular, it is known that obesity is associated with lower serum testosterone concentrations in men (Zumoff, 1988; Pasquali *et al*, 1991; Lima *et al*, 2000). Type II diabetes, which is related to obesity, was also observed to be associated with lower levels of testosterone (Giovannucci *et al*, 1998). Lower testosterone concentrations were in turn observed to be associated with an increased risk of aggressive tumours in two prospective cohort studies (Platz *et al*, 2005; Severi *et al*, 2006). Moreover, decreased levels of serum testosterone at PCa diagnosis were also observed to be associated with more aggressive tumours (Hoffman *et al*, 2000; Schatzl *et al*, 2001; D’Amico, *et al*, 2002; Massengill *et al*, 2003). It has been therefore hypothesised that lower serum testosterone levels may be associated with an increased risk of aggressive tumours and a decreased risk of the nonaggressive ones (Freedland and Platz, 2007; Hsing *et al*, 2007). Our results are in line with this hypothesis: a higher risk of advanced and fatal PCa and a lower risk of localised PCa among obese men. However, not all studies observed this relationship between aggressiveness of the tumour and serum testosterone levels (Fodstad *et al*, 2002).

The highest levels of IGF-I were observed in men with a BMI between ∼24 and 26 kg m^–2^ (Yamamoto and Kato, 1993; Kaaks and Lukanova, 2001; Lukanova *et al*, 2002). High IGF-I concentrations were observed to be directly associated with PCa incidence (Roddam *et al*, 2008). This would, at least partly, explain the highest incidence of localised PCa among men within the normal BMI range that we observed in our study and that was also observed among low-grade tumours in the Health Professionals Follow-up Study (Giovannucci *et al*, 2007). However, other studies observed a linear association between obesity and IGF-I concentrations (Nam *et al*, 1997; Kaaks *et al*, 2000). Furthermore, the association between IGF-I concentrations and type II diabetes is complex and it seems to be related to time since diagnosis – IGF-I may increase following insulin resistance and then decrease due to hypoinsulinaemia, as a result of damaged pancreatic *β-*cells (Bell and Polonsky, 2001).

It was suggested that physiologic changes during the years before age 30 may play an important role in the development of PCa (Hsing, 1996; Giovannucci *et al*, 1997). Obesity during adolescence, which was observed to persist in early adulthood (The *et al*, 2010), was also observed to be associated with delayed pubertal development (Wang, 2002). As puberty is associated with a steep increase in IGF-I (Keenan *et al*, 1993; Juul *et al*, 1994), a delay in this increase could mean a lower cumulative exposure to IGF-I and/or less exposure at crucial ages, and thus a possible reduced risk of PCa among men obese during early adulthood. This is in line with what we observed for advanced and fatal PCa.

The principal limitation of this study is the self-reported, questionnaire-based collection of current weight and weight at age 30 years and height, which are less accurate than anthropometric measures obtained directly by trained professionals. Nonetheless, self-reported current weight and height are shown to be highly correlated with measured weight and height in the Swedish adult population (Kuskowska-Wolk *et al*, 1989). However, some degree of nondifferential misclassification could have affected the recalled weight at age 30. Our study was observational and therefore we cannot completely exclude the possibility of residual confounding. Nevertheless, age-adjusted and multivariable-adjusted analyses provided overall similar estimates, suggesting that residual confounding is unlikely to explain totally our observed findings. As obesity has been observed to be associated with lower PSA values, and as in obese men the detection of PCa through digital rectal examination may be more complicated (Price *et al*, 2008), it is possible that some cases of incident PCa might have gone undetected among obese subjects, leading to detection bias. However, there is no national recommendation in Sweden for PSA-based PCa screening and the annual proportion of men aged 55–69 years who underwent a PSA test in the two study counties between 1997 and 2007 is estimated to be between 0% and 7% (Jonsson *et al*, 2011); thus, any bias introduced by PSA testing should be of limited relevance in our data.

The major strengths of this study include the relatively large size of the cohort, its population-based and prospective design, the relatively large number of incident PCa cases and the completeness of case ascertainment through the Regional and National Cancer Register. These study features substantially reduced the potential risk of selection bias and increased the generalisability of the study findings. As information on exposure was collected prospectively, any nondifferential misclassification would probably weaken rather than exaggerate the true relationship between body size and PCa incidence. Death from other causes than PCa could have impeded the study subjects to develop PCa, especially among obese and underweight men, thus leading to biased estimates. However, we observed only small differences when comparing results from the Cox proportional hazards with the competing-risks models, suggesting that our results were not affected by competing events.

In conclusion, we found some evidence that obesity in middle-aged and elderly men may decrease the risk of localised PCa. On the other hand, our results indicate that there might be a dual effect of obesity on advanced and fatal PCa: an inverse relationship for BMI at age 30 years and a direct relationship for BMI during middle-late adulthood. From a public health perspective, encouraging obesity is not a realistic way to reduce PCa morbidity. The biologic mechanisms behind the relationship between obesity and PCa incidence remain unclear, and thus replication of epidemiological studies and further work in understanding the underlying biologic mechanisms is necessary.

## Figures and Tables

**Table 1:** Age-standardised baseline characteristics by level of BMI at baseline age in the cohort of 36 959 Swedish men aged 45–79 years

	**BMI at baseline age, kg** **m**^–**2**^					
**Characteristics** a	**<21**	**21–22.9**	**23–24.9**	**25–27.4**	**27.5–29.9**	**⩾30**
No. of subjects	1863	5123	9551	11 244	5597	3581
Age at baseline (mean, years)	60	59	59	59	59	59
BMI at age 30 years (mean, kg m^–2^)	20	21	22	23	24	26
History of diabetes (yes, %)	4	5	7	12	18	31
Family history of prostate cancer (yes, %)	7	7	6	6	7	6
*Smoking status (%)*						
Current smoker	33	25	25	24	23	24
Former smoker	26	33	37	41	45	46
Never smoker	41	41	38	35	33	29
Physical activity (⩾45 MET-h per day, %)	20	21	21	20	18	16
Total energy intake (mean, kcal)	2813	2817	2773	2733	2720	2682
Years of education (>12 years, %)	24	23	20	17	14	12

Abbreviations: BMI=body mass index; MET=metabolic equivalent of task.

a All factors, except age and BMI at age 30 years, were directly standardised to the age distribution of the study participants. Percentages may not sum to 100 because of rounding.

**Table 2:** Rate ratios for incidence of total prostate cancer and its subtypes by levels of BMI at baseline age in the cohort of 36 959 Swedish men aged 45–79 years

	**BMI at baseline age (reference point), kg** **m^–2^**						
	**<21 (18)**	**21–22.9 (22)**	**23–24.9 (24)**	**25–27.4 (26.25)**	**27.5–29.9 (28.75)**	**⩾30 (35)**	**For every 5** **kg** **m**^–**2**^ **BMI increase**
*Localised prostate cancer*							
Age-adjusted model							
No. of cases/person-years	63/18 017	247/51 462	408/96 263	475/113 744	212/56 659	125/35 647	
RR (95% CI)^a^	0.77 (0.54–1.11)	1	0.99 (0.93–1.05)	0.93 (0.84–1.03)	0.85 (0.74–0.97)	0.65 (0.50–0.85)	—b
Multivariable model^c^							
No. of cases/person-years	62/17 487	245/50 419	401/94 253	467/111 322	204/55 507	124/34 885	
RR (95% CI)^a^	0.78 (0.54–1.13)	1	1.00 (0.94–1.06)	0.95 (0.86–1.05)	0.88 (0.76–1.02)	0.71 (0.53–0.94)	—b
RR (95% CI)^a^^,^^d^	0.70 (0.49–1.01)	1	1.01 (0.95–1.07)	0.97 (0.87–1.07)	0.89 (0.77–1.03)	0.69 (0.52–0.92)	—b
							
*Advanced prostate cancer*							
Age-adjusted model							
No. of cases/person-years	28/18 017	76/51 462	165/96 263	157/113 744	80/56 659	48/35 647	
RR (95% CI)^a^	1.01 (0.89–1.14)	1	1.00 (0.94–1.06)	0.99 (0.87–1.13)	0.99 (0.81–1.21)	0.98 (0.66–1.45)	0.99 (0.85–1.15)
Multivariable model^c^							
No. of cases/person-years	27/17 487	72/50 419	163/94 253	150/111 322	79/55 507	47/34 885	
RR (95% CI)^a^	0.97 (0.85–1.10)	1	1.02 (0.95–1.08)	1.03 (0.90–1.18)	1.05 (0.85–1.31)	1.11 (0.73–1.68)	1.04 (0.88–1.22)
RR (95% CI)^a^^,^^d^	0.96 (0.84–1.09)	1	1.02 (0.96–1.09)	1.05 (0.91–1.20)	1.07 (0.86–1.33)	1.15 (0.75–1.74)	1.05 (0.90–1.24)
							
*Fatal prostate cancer*							
Age-adjusted model							
No. of cases/person-years	11/16 931	35/48 500	62/90 692	61/106 984	31/53 086	25/33 396	
RR (95% CI)^a^	0.89 (0.74–1.07)	1	1.06 (0.97–1.16)	1.13 (0.93–1.38)	1.22 (0.89–1.67)	1.47 (0.81–2.69)	1.16 (0.92–1.46)
Multivariable model^b^							
No. of cases/person-years	11/16 426	35/47 524	62/88 804	59/104 705	29/51 989	23/32 679	
RR (95% CI)^a^	0.91 (0.75–1.11)	1	1.05 (0.95–1.16)	1.11 (0.89–1.36)	1.16 (0.83–1.63)	1.34 (0.70–2.55)	1.12 (0.87–1.43)
RR (95% CI)^a^^,^^d^	0.91 (0.75–1.10)	1	1.05 (0.95–1.15)	1.10 (0.90–1.36)	1.17 (0.85–1.62)	1.36 (0.73–2.53)	1.12 (0.88–1.43)

Abbreviations: CI=confidence interval; RR=rate ratio; BMI=body mass index.

a The RRs and 95% CIs were calculated in correspondence with the reference points.

b No RR for every 5 kg m^–2^ BMI at baseline age increase was calculated, as the relationship was modelled in a nonlinear fashion using second-degree fractional polynomials.

c Multivariable RRs were adjusted for BMI at age 30 years (kg m^–2^), age at baseline (years), total energy intake (kcal), total physical activity (<37.9, 38–40.9, 41–44.9, ⩾45 MET-h per day or missing), years of education (1–9, 9–12 or >12 years), smoking status (current, former or never smoker), family history of prostate cancer (yes, no or don’t know) and personal history of diabetes (yes or no).

d The RRs and 95% CIs calculated using competing-risks analysis. All the deaths from other causes than PCa were considered as competing events.

**Table 3:** Rate ratios for incidence of total prostate cancer and its subtypes by levels of BMI at age 30 years in the cohort of 36 959 Swedish men aged 45–79 years

	**BMI at age 30 years (reference point), kg** **m**^–**2**^						
	**<21 (18)**	**21–22.9 (22)**	**23–24.9 (24)**	**25–27.4 (26.25)**	**27.5–29.9 (28.75)**	**⩾30 (35)**	**For every 5** **kg** **m**^–**2**^ **BMI increase**
*Localised prostate cancer*							
Age-adjusted model							
No. of cases/person-years	290/67 977	550/120 481	472/116 166	161/49 565	42/11 629	15/5975	
RR (95% CI)^a^	1.01 (0.92–1.12)	1	0.99 (0.94–1.04)	0.99 (0.89–1.10)	0.98 (0.83–1.16)	0.96 (0.69–1.32)	0.98 (0.87–1.11)
Multivariable model^b^							
No. of cases/person-years	287/66 730	539/117 845	467/113 617	154/48 573	41/11 308	15/5800	
RR (95% CI)^a^	1.01 (0.91–1.12)	1	0.99 (0.94–1.05)	0.99 (0.89–1.10)	0.98 (0.82–1.16)	0.96 (0.69–1.34)	0.98 (0.87–1.12)
RR (95% CI)^a^^,^^c^	1.03 (0.93–1.14)	1	0.99 (0.94–1.04)	0.97 (0.87–1.08)	0.96 (0.81–1.14)	0.92 (0.66–1.28)	0.97 (0.85–1.10)
							
*Advanced prostate cancer*							
Age-adjusted model							
No. of cases/person-years	112/67 977	192/120 481	166/116 166	70/49 565	9/11 629	5/5975	
RR (95% CI)^a^	1.09 (0.93–1.28)	1	0.96 (0.88–1.04)	0.91 (0.77–1.08)	0.87 (0.66–1.14)	0.76 (0.45–1.28)	0.90 (0.73–1.10)
Multivariable model^b^							
No. of cases/person-years	108/66 730	185/117 845	164/113 617	69/48 573	8/11 308	4/5800	
RR (95% CI)^a^	1.09 (0.92–1.29)	1	0.96 (0.88–1.04)	0.91 (0.77–1.09)	0.87 (0.65–1.15)	0.76 (0.44–1.30)	0.90 (0.73–1.11)
RR (95% CI)^a^^,^^c^	1.11 (0.94–1.31)	1	0.95 (0.87–1.03)	0.90 (0.75–1.07)	0.84 (0.63–1.11)	0.71 (0.41–1.23)	0.88 (0.71–1.08)
							
*Fatal prostate cancer*							
Age-adjusted model							
No. of cases/person-years	50/64 052	78/113 654	69/109 099	22/46 355	4/10 879	2/5553	
RR (95% CI)^a^	1.31 (1.02–1.701)	1	0.87 (0.77–0.99)	0.75 (0.57–0.98)	0.63 (0.41–0.97)	0.41 (0.18–0.94)	0.71 (0.52–0.98)
Multivariable model^b^							
No. of cases/person-years	49/62 868	75/111 148	68/106 729	22/45 419	3/10 579	2/5384	
RR (95% CI)^a^	1.28 (0.99–1.67)	1	0.88 (0.77–1.01)	0.77 (0.58–1.02)	0.66 (0.42–1.03)	0.45 (0.19–1.05)	0.73 (0.53–1.02)
RR (95% CI)^a^^,^^c^	1.30 (1.00–1.71)	1	0.88 (0.77–1.00)	0.75 (0.57–1.01)	0.64 (0.41–1.01)	0.42 (0.18–1.02)	0.72 (0.51–1.01)

Abbreviations: CI=confidence interval; RR=rate ratio; BMI=body mass index.

a The RRs and 95% CIs were calculated in correspondence with the reference points.

b Multivariable RRs were adjusted for BMI at age 30 years (kg m^–2^), age at baseline (years), total energy intake (kcal), total physical activity (<37.9, 38–40.9, 41–44.9, ⩾45MET-h per day or missing), years of education (1–9, 9–12 or >12 years), smoking status (current, former or never smoker), family history of prostate cancer (yes, no or don’t know) and personal history of diabetes (yes or no).

c RRs and 95% CIs calculated using competing-risks analysis. All the deaths from other causes than PCa were considered as competing events.
